# A Bayesian Approach to Modeling Risk of Hospital Admissions Associated With Schizophrenia Accounting for Underdiagnosis of the Disorder in Administrative Records

**DOI:** 10.1162/CPSY_a_00010

**Published:** 2018-02-01

**Authors:** Eileen M. Stock, James D. Stamey, John E. Zeber, Alexander W. Thompson, Laurel A. Copeland

**Affiliations:** 1 Cooperative Studies Program Coordinating Center, VA Maryland Health Care System, Departmentof Veterans Affairs, Perry Point, Maryland, USA; 2 Center for Applied Health Research, Central Texas Veterans Health Care System/Baylor Scottand White Health, Temple, Texas, USA; 3 Texas A&M Health Science Center, Bryan, Texas, USA; 4 Department of Statistical Science, Baylor University, Waco, Texas, USA; 5 Department of Psychiatry, University of Iowa Carver College of Medicine, Iowa City, Iowa, USA

**Keywords:** schizophrenia, underdiagnosis, Bayesian analysis, measurement error

## Abstract

Schizophrenia is a debilitating serious mental illness characterized by a complex array of symptoms with varying severity and duration. Patients may seek treatment only intermittently, contributing to challenges diagnosing the disorder. A misdiagnosis may potentially bias and reduce study validity. Thus we developed a statistical model to assess the risk of 1-year hospitalization for patients diagnosed with schizophrenia, accounting for when schizophrenia is underreported in administrative databases. A retrospective study design identified patients seeking care during 2010 within an integrated health care system from the Health Maintenance Organization Research Network located in the southwestern United States. Bayesian analysis addressed the problem of underdiagnosed schizophrenia with a statistical measurement error model assuming varying rates of underreporting. Results were then compared to classical multivariable logistic regression. Assuming no underreporting, there was an 87% greater relative odds of hospitalization associated with schizophrenia, OR = 1.87, CI [1.08, 3.23]. Effect sizes and interval estimates representing the association between hospitalization and schizophrenia were reduced with the Bayesian approach accounting for underdiagnosis, suggesting that less severe patients may be underrepresented in studies of schizophrenia. The analytical approach has useful applications in other contexts where the identification of patients with a given condition may be underreported in administrative records.

## INTRODUCTION

Schizophrenia is a debilitating serious mental illness (SMI), with a prevalence of approximately 1% in all cultures (Kelly, Conley, & Carpenter, 2005; Schultz, North, & Shields, 2007). Although minimal prevalence differences exist, variability in the diagnosis of schizophrenia still persists (Blow et al., 2004). Misdiagnosis may arise from the varied presentation of a multitude of symptoms associated with the disorder, often overlapping with bipolar disorder or schizo affective disorder (Blow et al., 2004). The diagnostic process must take into consideration the patient’s subjective experiences in multiple cognitive domains as well as his or her behavior and functioning in various environments. Correct diagnosis can be difficult, especially during initial onset of symptoms. Clinicians often must monitor the patient to establish recurrence, chronicity, and intensity, which may take months or years (Altamura & Goikolea, 2008; Ries, Bokan, & Schuckit, 1980).

Inconsistency in the diagnostic criteria defining schizophrenia continues to pose significant challenges to estimating prevalence (McCormick & Flaum, 2005; Tandon, Keshavan, & Nasrallah, 2008). The need for expert judgment in the diagnostic process introduces a source of uncertainty (Rendall, Handcock, & Jonsson, 2009; Saha, Chant, Welham, & McGrath, 2005; Tandon et al., 2008). In one study of diagnostic uncertainty, among 254 psychiatric inpatients, 6% were diagnosed with schizophrenia and 12% narrowly missed the definite diagnosis but were later suspected to have the disorder (Ries et al., 1980). In addition, misdiagnosis may lead to these individuals’ exclusion from studies of course of illness and treatment outcomes, potentially biasing a study’s findings and weakening its conclusions. Many administrative database-only studies rely on ICD-9 diagnosis codes for the identification of patients with schizophrenia, omitting persons without the designated codes in their electronic data. However, this limitation can possibly be mitigated with advanced analysis methods, including the application of Bayesian techniques to account for beliefs about the rate of misdiagnosis or underreporting of schizophrenia.

Multiple factors may increase the likelihood of hospital admission among individuals with schizophrenia. A misdiagnosis during initial onset may result in ineffective treatment that can exacerbate symptoms (Altamura & Goikolea, 2008; Blow et al., 2004). Furthermore, once diagnosed, some individuals with schizophrenia may not pursue treatment (owing to, e.g., stigma, fear of loss of work, lack of insight; Marwaha & Johnson, 2004) or may do so only intermittently (Tsan et al., 2012). For those in care, compliance with treatment recommendations is low, with approximately 50%–65% adherence to psychiatric outpatient care or medica tion (Altamura & Goikolea, 2008; Lang, Federico, Muser, & Menzin, 2013; Olfson, Mechanic, Boyer, & Hansell, 1998; Valenstein et al., 2004). Treatment nonadherence increases the likelihood of acute exacerbation and rehospitalization (Altamura & Goikolea, 2008; Valenstein et al., 2002). In fact, the annual rate of all-cause admission among patients diagnosed with schizophrenia can reach 29% (Lang et al., 2013; Olfson et al., 1998).

The underreporting, or hereinafter simply underdiagnosis, of schizophrenia in studies involving patients with the disorder may potentially bias results and reduce the validity of research findings. Underdiagnosis introduces a source of uncertainty that is difficult to address within the classical frequentist statistical paradigm (McMillan, Bedrick, & C’DeBaca, 2009; Rendall et al., 2009). Thus we propose a Bayesian approach to the problem of underdiagnosed schizophrenia through the use of a statistical measurement error model that explicitly evaluates the impact of varying assumptions about the extent of underreporting. This approach is similar to that used by McMillan et al. (2009) on the effects of drug use when self-reported behavior may be underreported and by MacLehose et al. (2009) on correcting for misclassification when self-reported maternal smoking is underreported. Similarly, we present a framework for estimating the effects of “true” schizophrenia when the disorder might be underdiagnosed and its true associated risk for hospitalization accounting for uncertainty in the diagnosis process. In this study, we compare the odds of 1-year all-cause hospital admission among patients with schizophrenia to patients with and without other SMIs (bipolar disorder, posttraumatic stress disorder [PTSD], and major depressive disorder [MDD]), accounting for uncertainty regarding schizophrenia diagnosis. By accounting for this uncertainty, improved estimates of the association between schizophrenia and all-cause admission are anticipated.

## METHODS

### Study Sample

A retrospective study design was employed utilizing data on patients seeking care in an integrated health care system located in the southwestern region of the United States. The project was approved by the local institutional review board prior to study initiation. The study site represented 1 of 19 care-and-coverage health systems across the United States composing the Health Maintenance Organization Research Network (HMORN) Virtual Data Warehouse (Go et al., 2008). The HMORN develops a uniform set of health care measures from member data, primarily health care claims, to conduct population health services research (Copeland & Zeber, 2013; Ross et al., 2014; Stevens & Sanghi, 2010). The year 2010 served as the baseline year. Additional criteria required that patients be at least 18 years of age and enrolled in the system’s health plan 1 year prior for assessing baseline characteristics and 1 year post for measuring study outcomes. A total of 87,806 patients were identified.

### Measures

Patient demographic measures included age, gender, race, Hispanic ethnicity, and mental and physical comorbidities. Race had categories of White, Black, and other/missing race. Patients with SMIs were identified from administrative diagnosis codes, hierarchically defined as schizophrenia (ICD-9 code 295, excluding 295.5 latent), bipolar disorder (296.0, 296.1, 296.4–296.8), PTSD (309.81), and MDD (296.2, 296.3, 311). Physical comorbidity was captured using the Selim Physical comorbidity index, which sums 30 chronic medical conditions (range, 0–30) extracted from inpatient and outpatient records in the year prior (Copeland et al., 2009; Pugh et al., 2005; Selim et al., 2004). The primary outcome of interest was all-cause hospitalization (psychiatric or medical) during a 1-year follow-up period per claims data on admissions to any of the health care system’s hospitals.

### Analysis Plan

Bivariate analyses compared patient characteristics by SMI status (schizophrenia, bipolar disorder, PTSD, MDD, and non-SMI), chi-square analyses for categorical variables, and the Kruskal–Wallis test for continuous variables. The multivariable models included adjustment for suspected underdiagnosis, as described subsequently.

### Statistical Model

To construct a measure of uncertainty regarding underdiagnosed schizophrenia, we estimated the degree of underdiagnosis from available information. One-year prevalence of schizophrenia at our site was 0.13%, lower than the national average of 0.5% (5.1 per 1,000 persons; Wu, Shi, Birnbaum, Hudson, & Kessler, 2006). Because schizophrenia is a major risk factor for homelessness, with up to 20% homeless in a 1-year period, these individuals are less likely to engage in mental health or medical treatment (Folsom et al., 2005; Foster, Gable, & Buckley, 2012). Assuming 20% of persons with schizophrenia are homeless, and therefore have no record of their diagnosis in our data for the study year, the annual prevalence of schizophrenia among individuals seeking treatment is estimated at 0.4% (80% of 0.5%). Assuming that only 50% of these patients comply with psychiatric treatment and attend visits (Olfson et al., 1998), we anticipated an observable 1-year prevalence for schizophrenia of approximately 0.2% (50% of 0.4%) in our health care system.

To implement the Bayesian approach to the problem of covariate measurement error for a binary explanatory variable, we specified the 1-year prevalence of schizophrenia in the study population as 0.20% and the rate of underdiagnosis as 0.20% minus 0.13% (our system’s documented prevalence rate), or 0.07%. The relationship between schizophrenia and admission was described with a Bayesian logistic regression model. The outcome of admission, denoted *A*_*i*_ for the *i*th patient, was assigned a Bernoulli distribution (McMillan et al., 2009). Then, for the explanatory variable denoting the *true* presence of schizophrenia, denoted *D*_*i*_, we assigned *D*_*i*_ = 1 for patients *truly* with the disorder of schizophrenia and *D*_*i*_ = 0 otherwise. Additional covariates were included in the model for the purpose of comparing rates of admission across SMIs (schizophrenia vs. bipolar, PTSD, and MDD) and adjusting for baseline patient differences to control for potential confounding (decade effect of age, gender, race/ethnicity, and Selim Physical). Letting *X*_*i*_ be a vector of length *J* containing covariate responses for the *i*th patient, admission was modeled as

$$A_{i}∼\text{Bernoulli}\left(p_{i}\right)$$

$$\text{logit}\left(p_{i}\right)={\;\beta }_{0}{+\beta }_{1}D_{i}+\;\beta^{\prime }{\text{X}}_{i},$$

where *p*_*i*_ is the 1-year probability of a hospital admission, β_0_ is the intercept, β_1_ is the regression coefficient corresponding to *truly* having schizophrenia describing the effect of the disorder on admission, and the vector ***β*** of length *J* contains the regression coefficients for the remaining covariates. In this model, β_1_ measures the relative increase in the log odds of admission associated with *true* schizophrenia.

The *reported* diagnosis of schizophrenia per clinician examination and medical utilization records for the *i*th patient, denoted as *R*_*i*_, was assumed to have a Bernoulli distribution conditioned on truly having the disorder (McMillan et al., 2009):

$$R_{i}|\;D_{i}\;∼\text{Bernoulli}\left[\left(1-\lambda \right)\;D_{i}\right],$$

where λ is the rate at which schizophrenia is underdiagnosed and $s=\left(1-\lambda \right)$ is the sensitivity of diagnosing schizophrenia. The probability of correctly diagnosing schizophrenia through clinical assessment and ICD-9 diagnosis codes is zero if the patient truly does not have the disorder and $\left(1-\lambda \right)$ otherwise:

$$P(R_{i}\;|\;D_{i})=\left\{\begin{array}{l} 0,\;\text{ if}\;\;D_{i}=0\;\\ s,\;\text{ if}\;\;D_{i}=1, \end{array}\right.$$

assuming overdiagnosis of schizophrenia is not present. The true, unobserved occurrence of schizophrenia is assumed to be a Bernoulli random variable,

$$D_{i}\;∼\text{Bernoulli}\left(\theta \right),$$

with 1-year period prevalence θ.

### Prior Specification

In the logit model, the coefficients, β_1_ and ***β****′*, were assigned a relatively noninformative normal prior distribution with mean zero and a large variance:

$$\beta_{i}\;∼\text{Normal}\left(0,10,000\right)\;,i=1,\ldots ,n,$$

where *n* = 10 denotes the number of nonintercept model coefficients. The intercept, β_0_, was given a normal prior distribution centered at −3, resembling that observed in preliminary analyses, and a variance of 10 to allow for enough variability to incorporate the possibility of various other intercepts:

$$\beta_{0}∼\text{Normal}\left(-3,10\right).$$

Knowledge about the actual prevalence of schizophrenia, θ, and the sensitivity of diagnosing the condition, *s*, can be incorporated through specification of the parameters’ prior distributions. Using our described approach, we expected to observe a period prevalence of 0.2% based on ICD-9 diagnosis codes in the medical records. This corresponds to a low sensitivity of 65% (0.13%/0.20%). If the true prevalence of schizophrenia among the study population consisting of treatment-seeking patients was about one-fourth less than that expected, say, 0.15%, then the sensitivity of diagnosing the disorder is believed to be high, estimated at 87% (0.13%/0.15%), and for a period prevalence of 0.17%, a moderate sensitivity of 76% (0.13%/0.17%) is observed. The sensitivities of 65%, 76%, and 87% correspond to 1-year underdiagnosis rates of 35%, 24%, and 13%, respectively. Although 35% is fairly high (one in three patients with schizophrenia is not diagnosed with the disorder), 24% is a more moderate estimate (one in four), and 13% (one in eight) denotes a low rate of underdiagnosing. Three different scenarios were considered, each corresponding to a different sensitivity.

Uncertainty about the period prevalence of schizophrenia was estimated with an informative independent beta prior distribution constructed by directly matching the sensitivity from each scenario to the mean of the beta distribution (Joseph, Gyorkos, & Coupal, 1995). When there is a priori knowledge that the prevalence, θ, is small, the class of Beta(1,α) prior distributions is considered more appropriate (Pritchard & Tebbs, 2011). Period prevalence rates of 0.20%, 0.17%, and 0.15% were examined in this study, exploring the sensitivity of the model to prior beliefs. Uncertainty about the sensitivity of diagnosing schizophrenia was estimated with a mildly informative independent beta prior distribution for the three prevalence scenarios considered: (a) low sensitivity (θ = 0.20*%*, *s* = 65*%*) : θ ∼ Beta(1,499), *s* ∼Beta(19.5,10.5); (b) moderate sensitivity (θ = 0.17*%*, *s* = 76*%*) : θ∼Beta(1,587), *s* ∼ Beta(22.8,7.2); and (c) high sensitivity (θ = 0.15*%*, *s* = 87*%*) :θ∼Beta(1,665), *s* ∼Beta(26.1,3.9). The sample size equivalent of the prior information for the diagnosing sensitivity is set to 30, less than that used in other studies, and is therefore considered mildly informative (Dendukuri, Rahme, Belisle, & Joseph, 2004; Joseph et al., 1995). To reflect prior beliefs, the period prevalence was assigned more informative prior distributions.

In the Bayesian framework, expert knowledge and parameter constraints can be easily incorporated through prior specification. Another advantage is that the methods do not rely on asymptotic assumptions and thus do not require large samples for analyses. The number of parameters to be estimated is not limited by the number of observations. Evaluation of the posterior distribution was implemented through simulation using Markov chain Monte Carlo techniques in WinBUGS, Version 1.4.3 (Medical Research Council and Imperial College, UK). The code used for the model of the underdiagnosis of schizophrenia is shown in Stock, Stamey, Zeber, Thompson, and Copeland (2017, Appendix). Two chains were initialized with a 10,000 run burn-in and 50,000 sample updates. Following convergence of the chains, approximate 95% credible intervals (Bayesian probability intervals) for model parameters of each scenario (represented as odds ratios) were obtained and compared to those of the classical logistic regression model, not accounting for the bias and uncertainty due to the underreporting of schizophrenia. To summarize, the Bayesian methodological approach takes into account the observed rate of schizophrenia from administrative diagnosis codes and various assumptions about treatment and homelessness and then applies a sensitivity analysis technique to gauge the effect of underdiagnosis on an important clinical outcome (i.e., admissions).

## RESULTS

Among 87,806 patients receiving care in 2010, 57% were female, 71% were White, and 5% were Hispanic, with a median age of 53 years (Table 1). In this cohort, 6,401 (7.3%) patients were diagnosed with a SMI, where 114 (1.3 per 1,000 patients) were diagnosed with schizophrenia, 412 (4.7 per 1,000) with bipolar disorder, 82 (0.9 per 1,000) with PTSD, and 5,793 (66.0 per 1,000) with MDD. Patients with bipolar disorder or PTSD comprised the youngest patients (median age 48 years), whereas patients with schizophrenia were older (median age 55 years), χ_0_(4, *N* = 87,806) = 61.8, *p* < 0.01. Across SMI categories, women more commonly had MDD (74% vs. 55%–62%), χ_0_(4, *N* = 87,806) = 728.2, *p* < 0.01. Black patients were diagnosed with schizophrenia at a higher rate than they were with other SMIs (15% vs. 3%–9%), χ_0_(4, *N* = 87,806) = 50.4, *p* < 0.01, whereas Hispanic patients were least likely to be diagnosed with schizophrenia (2% vs. 4%–6%), χ_0_(4, *N* = 87,806) = 10.8, *p* = 0.03. One-year hospital admission was greatest among patients with schizophrenia (14%), followed by other SMIs (12%–13%), and was lowest among non-SMI patients (8%), χ_0_(4, *N* = 87,806) = 106.4, *p* < 0.01.

**Table 1.** Patient characteristics, overall and by SMI status (*N* = 87,806).

**Variable**	**Total (*N* = 87,806)**	**Schizophrenia (*N* = 114)**	**Bipolar (*N* = *412)***	**PTSD (*N* = 82)**	**MDD (*N* = 81,405)**	**Non-SMI (*N* = 5,793)**	**Test statistic**	***p* Value^*a*^**
Age								
*Mean (SD):*	52.7 (18.9)	52.7 (19.8)	47.3 (17.4)	47.0 (16.6)	51.7 (18.1)	52.8 (19.9)	61.8	<0.01
*Median (min–max):*	53 (18–103)	55 (18–89)	48 (18–89)	48 (18–87)	51 (18–98)	53 (18–103)		
Female (%)	50,359 (57.4)	63 (55.3)	256 (62.1)	47 (57.3)	4,300 (74.2)	45,693 (56.1)	728.2	<0.01
Race (%)							155.7	<0.01
White	62,394 (17.1)	80 (70.2)	319 (77.4)	57 (69.5)	4,493 (77.6)	57,445 (70.6)	136.9	<0.01
Black	5,816 (6.6)	17 (14.9)	13 (3.2)	7 (8.5)	285 (4.9)	5,494 (6.7)	50.4	<0.01
Other	19,596 (22.3)	17 (14.9)	80 (19.4)	18 (22.0)	1,015 (17.5)	18,466 (22.7)	88.8	<0.01
Hispanic (%)	4,763 (5.4)	2 (1.8)	15 (3.6)	5 (6.1)	277 (4.8)	4,464 (5.5)	10.8	0.03
							
Selim								
*Mean (SD):*	1.7 (1.8)	1.7 (1.8)	1.7 (1.8)	1.9 (2.1)	2.0 (2.1)	1.7 (1.8)	122.3	<0.01
*Median (min–max):*	1 (0–15)	1 (0–9)	1 (0–8)	1 (0–14)	1 (0–15)	1 (0–15)		
Admission (%)	7,269 (8.3)	16 (14.0)	53 (12.9)	10 (12.2)	668 (11.5)	6,522 (8.0)	106.4	<0.01


^*a*^Patient characteristics were compared across SMI groups by employing Chi-square analyses for categorical variables and the Kruskal-Wallis test for continuous variables, assuming a Type I error of *α* = 0.05.

Assuming underdiagnosis of schizophrenia is absent from the administrative data (naive model), there was an 87% greater relative odds of admission associated with schizophrenia, OR (odds ratio) = 1.87, CI (confidence interval) [1.08, 3.23] (Table 2) observed in the classical logistic regression model; 73% for bipolar disorder, OR = 1.73, CI [1.28, 2.32]; 28% with MDD, OR = 1.28, CI [1.17, 1.40]; and no association with PTSD, OR = 1.52, CI [0.76, 3.05] (n.s.). Older age, being female, and having comorbid physical conditions were each associated with greater odds of 1-year admission.

**Table 2.** Bayesian logistic regression model results for varying sensitivities, along with the classical logistic regression model approach

	**Classical model (logistic regression)**		**Low sensitivity (*θ* = 0.20%, *s* = 65%)**		**Moderate sensitivity (*θ* = 0.17%, *s* = 76%)**		**High sensitivity (*θ* = 0.15%, *s* = 87%)**	
**Predictor**	**OR**	**95% CI**	**OR**	**95% CI**	**OR**	**95% CI**	**OR**	**95% CI**
Schizophrenia	1.87	[1.08, 3.23]	1.83	[1.02, 3.12]	1.82	[1.02, 3.09]	1.82	[1.02, 3.10]
Bipolar disorder	1.73	[1.28, 2.32]	1.71	[1.26, 2.29]	1.71	[1.26, 2.29]	1.71	[1.26, 2.29]
PTSD	1.52	[0.76, 3.05]	1.46	[0.69, 2.85]	1.46	[0.68, 2.86]	1.46	[0.69, 2.84]
MDD	1.28	[1.17, 1.40]	1.28	[1.17, 1.40]	1.28	[1.17, 1.40]	1.28	[1.17, 1.40]
Age (decade effect)	1.03	[1.01, 1.05]	1.03	[1.01, 1.05]	1.03	[1.01, 1.05]	1.03	[1.01, 1.05]
Female	1.31	[1.25, 1.38]	1.31	[1.25, 1.38]	1.31	[1.25, 1.38]	1.31	[1.25, 1.38]
Black race	1.07	[0.97, 1.18]	1.07	[0.97, 1.18]	1.07	[0.97, 1.18]	1.07	[0.97, 1.18]
Other race	0.83	[0.77, 0.89]	0.83	[0.77, 0.89]	0.83	[0.77, 0.89]	0.83	[0.77, 0.89]
Hispanic	1.09	[0.98, 1.22]	1.09	[0.97, 1.22]	1.09	[0.97, 1.22]	1.09	[0.97, 1.22]
Selim Physical	1.35	[1.34, 1.37]	1.35	[1.34, 1.37]	1.35	[1.34, 1.37]	1.35	[1.34, 1.37]


*Note.* *N* = 87,806. CI = confidence interval (credible for Bayesian).

A Bayesian analysis for each scenario of varying underdiagnosis rates for schizophre nia (sensitivities of *s* = 65%, 76%, and 87%) was performed. Results of the Bayesian logistic regression models are reported in Table 2. Convergence of estimates was observed for all models. Posterior density plots and trace plots are available from the authors on request. In the Bayesian analyses assuming varying sensitivities, all effect sizes representing the true diagnosis of schizophrenia were smaller to that observed with the classical approach. The relative odds of admission associated with schizophrenia were 4%–5% lower compared to the naive model. Similarly, the relative odds of admission associated with bipolar disorder and PTSD was 2% and 6% lower, respectively, compared to the naive model. Although a mildly informative prior for the intercept was used, similar performance was observed with a more diffuse prior, Normal(0,10,000).

In the Bayesian models, 95% credible interval lengths corresponding to the estimates for schizophrenia, bipolar disorder, and PTSD were each smaller than the resulting 95% confidence intervals observed with the classical approach. In the Bayesian analyses, the 95% credible interval lengths ranged from 1.82 to 1.83, depending on the sensitivity scenario considered, versus 1.87 in the naive model. This was also true for bipolar disorder (1.71 vs. 1.73) and PTSD (2.15–2.18 vs. 2.29). Estimates and interval lengths for MDD and other covariates were similar across models in both the frequentist and Bayesian frameworks.

## DISCUSSION

When studies examine health outcomes or associated risks in persons with schizophrenia, findings would be enhanced by a diagnosis that is accurate and has been made for all persons in the sampling frame. Otherwise, results may be biased depending on whether there is a tendency to underdiagnose or overdiagnose the disorder. Imagine, for example, a study examining all-cause hospitalization comparing those with schizophrenia to those with bipolar disorder. If, in the total population, the true prevalence rate of schizophrenia is 1%, though it has only been diagnosed in 0.5%, the results may under- or overstate the risk of hospitalization in those with schizophrenia. We have examined a statistical method to help address this problem in mental health services research. In our study, the risk of hospitalization for patients with schizophrenia was overstated when not accounting for underdiagnosis of the disorder in administrative records.

The proposed Bayesian models provided a means to examine the relationships between schizophrenia and 1-year hospitalization under a variety of prior assumptions about the prevalence of schizophrenia in the target population and the rate of underdiagnosis in the administrative data. The Bayesian approach demonstrated that the effects of the mental disorder on admission are smaller in magnitude than those observed with a classical approach assuming no underdiagnosis of the disorder per ICD-9 diagnosis codes. It has been well documented that the characteristics of a community, such as race and economic status, can largely influence the reported prevalence rate of schizophrenia (Saha et al., 2005; Tandon et al., 2008). Consequently, the effects of schizophrenia on admission rates when underdiagnosed that we observed here may not be generalizable to other health care systems suspecting an underdiagnosis of the disorder. Improvements in the diagnosis of mental disorders are being made all the time, but much work remains to be done (Mewton, Slade, Teesson, Memedovic, & Krueger, 2014). The proposed model accounting for the underreporting of schizophrenia demonstrates its utility and applicability to account for the underdiagnosis of other disorders.

The proposed model assumes that there is no overdiagnosis of schizophrenia, only underdiagnosis. Accounting for both imperfect sensitivity and imperfect specificity would be straightforward (Dendukuri et al., 2004; Joseph et al., 1995) but would add an extra parameter that would require an informative prior. This could add an unnecessary complication in cases where specificity is very close to 1. We believe this is a reasonable assumption given that cli nicians tend to delay the diagnosis to establish recurrence, chronicity, and intensity (Altamura & Goikolea, 2008; Ries et al., 1980). Thus a diagnosis of schizoaffective disorder (ICD-9 code 295.70) is often used, a code we included in identifying patients with schizophrenia. In some health care systems, the unspecified psychotic disorder ICD-9 code 298.9 is also included in defining the diagnosis of schizophrenia, as it may be used alternatively to the 295 codes (Sun et al., 2014). We assessed this code in our own health care system but still found the 1-year period prevalence of schizophrenia to be less than the national average. A limitation is that a large proportion of the studied insured group is employed in the health care system, which could bias the sample toward the working well. Additionally, prevalence rates may vary for individuals with schizophrenia being treated acutely versus those seeking long-term treatment.

Interestingly, this study found that Hispanic patients were less likely to be diagnosed with schizophrenia than with other SMIs, which varies from previous literature on veterans (Blow et al., 2004). This may be the result of regional or institutional differences that should be examined further. An extension of the proposed Bayesian approach could be to consider characteristics in the exposure model or account for under- and overrepresentation of other covariates in the disease model.

While we should always seek to represent a disorder more accurately in studies, our findings suggest that using ICD-9 codes for the diagnosis of schizophrenia does not introduce serious bias. In our system, we found the impact of underdiagnosis of schizophrenia on at least one key outcome (admissions) to vary little under different assumptions. Thus, regardless of many potential sources of diagnosis complexity or errors, we can place greater confidence in utilizing administrative diagnosis codes for identifying patients with schizophrenia in mental health services research. After attempting to more accurately represent the diagnosis of schizophrenia and estimate the 1-year risk of admission, our findings imply that the misdiagnosis we suspected was not a major influence. However, these findings may vary across health care systems and for other diagnoses.

## CONCLUSION

Many studies rely on ICD-9 diagnosis codes for the identification of patients with schizophrenia when assessing health outcomes in administrative data. However, the complex spectrum of schizophrenia symptoms, their varying severity and duration, can make it difficult to correctly diagnose the disorder, and many patients may narrowly miss the clinical criteria for diagnosis, especially during onset of the disease. An incorrect or delayed diagnosis can lead to inappropriate treatment, symptom exacerbation, and worse outcomes, such as hospitalization. In this article, we investigated the impact of underdiagnosing schizophrenia on analyses of risk factors for hospitalization using a Bayesian approach. We observed reduced correlation between hospitalization and diagnosis of schizophrenia, as well as other mental disorders, uniformly across varying rates of underdiagnosis. Although effect sizes may vary across health care systems, we believe the analytical approach has useful applications in studies relying on administrative records for the identification of patients with schizophrenia or other conditions subject to underdiagnosis.

## AUTHOR CONTRIBUTIONS

Eileen M. Stock and James D. Stamey conceptualized the study. Eileen M. Stock conducted analyses. Eileen M. Stock, James D. Stamey, John E. Zeber, Alexander W. Thompson, Laurel A. Copeland contributed to the interpretation of the results and to the writing of the article.

## FUNDING INFORMATION

This work was supported by the Center for Applied Health Research, a research center jointly sponsored by Central Texas Veterans Health Care System and Baylor Scott and White Health in Temple, Texas. The views expressed in this article are those of the authors and do not necessarily reflect the position or policy of the Department of Veterans Affairs or the United States.
